# Pinostilbene inhibits lung epithelial-mesenchymal transition and delays pulmonary fibrosis by modulating the PI3K/Akt pathway

**DOI:** 10.3389/fphar.2025.1614546

**Published:** 2025-09-05

**Authors:** Xin Yu, Li Xiao, Jiang Zhu, Tianying Sun, Kai Gong, Xuefang Kou, Yuhe Zhou, Mengzhen Xu, Kaihui Lu, Hua Sun, Haixing Guan, Chuanguo Liu

**Affiliations:** ^1^ Innovative Institute of Chinese Medicine and Pharmacy, Shandong University of Traditional Chinese Medicine, Jinan, China; ^2^ Department of Orthopedics, Affiliated Hospital of Shandong University of Traditional Chinese Medicine, Jinan, China; ^3^ Experimental Center, Shandong University of Traditional Chinese Medicine, Jinan, China; ^4^ Department of Radiation Oncology Physics and Technology, Shandong Cancer Hospital and Institute, Shandong First Medical University and Shandong Academy of Medical Sciences, Jinan, China; ^5^ Key Laboratory of Traditional Chinese Medicine Classical Theory, Ministry of Education, Shandong University of Traditional Chinese Medicine, Jinan, China

**Keywords:** pinostilbene, epithelial-mesenchymal transition, pulmonary fibrosis, PI3K/Akt signaling pathway, TGF-β1

## Abstract

Epithelial-mesenchymal transition (EMT) in the lung is a key process in which pulmonary epithelial cells lose epithelial characteristics and acquire mesenchymal properties, contributing to conditions such as pulmonary fibrosis. This study investigates the potential of pinostilbene (PIN), a natural stilbene compound with known anti-cancer, antioxidant and anti-inflammatory properties, to inhibit pulmonary EMT. Cellular experiments using A549 and Beas2B cells showed that PIN significantly reduced TGF-β1-induced mesenchymal marker expression while increasing epithelial marker expression. Functional assays confirmed the ability of PIN to inhibit cell migration and adhesion. *In vivo*, PIN alone or in combination with pirfenidone effectively alleviated lung damage in a murine lung fibrosis model, as demonstrated by histological analysis. Mechanistic studies identified the PI3K/Akt pathway as a target of PIN, with Western blot analysis showing decreased phosphorylation levels of PI3K and Akt. These findings suggest that PIN inhibits pulmonary EMT and delays the progression of pulmonary fibrosis by modulating the PI3K/Akt pathway, providing a promising therapeutic avenue for lung diseases associated with EMT.

## 1 Introduction

Pulmonary EMT represents a sophisticated cellular biological process during which pulmonary epithelial cells forfeit their epithelial traits and assume a mesenchymal phenotype. Epithelial cells typically exhibit polarity, form organized layers through tight junctions and adherens junctions, and express epithelial markers such as E-cadherin. However, upon undergoing EMT, cells lose their polarity, intercellular junctions weaken, the expression of tight junction proteins is reduced, and the expression of mesenchymal markers like Vimentin and α-SMA is initiated. The morphology of the cells shifts from a polygonal epithelial pattern to a spindle-shaped mesenchymal pattern, with a concurrent increase in cellular migration and invasiveness (Wang and Hsu, 2024). Transforming growth factor-beta 1 (TGF-β1) plays an extremely crucial role in inducing the process of pulmonary EMT (Zhang L. B. et al., 2023). Upon binding to receptors on pulmonary epithelial cells, TGF-β1 activates intracellular signaling pathways that downregulate E-cadherin expression and promote transcription factors like Snail and Slug. These factors, in turn, further repress E-cadherin and activate the expression of mesenchymal marker genes, including Vimentin and α-SMA, thus promoting the transformation of pulmonary epithelial cells into mesenchymal cells (Zhang L. et al., 2023). Pulmonary EMT is critically involved in the pathogenesis and progression of various pulmonary diseases, including pulmonary fibrosis and lung cancer.

**FIGURE 1:**
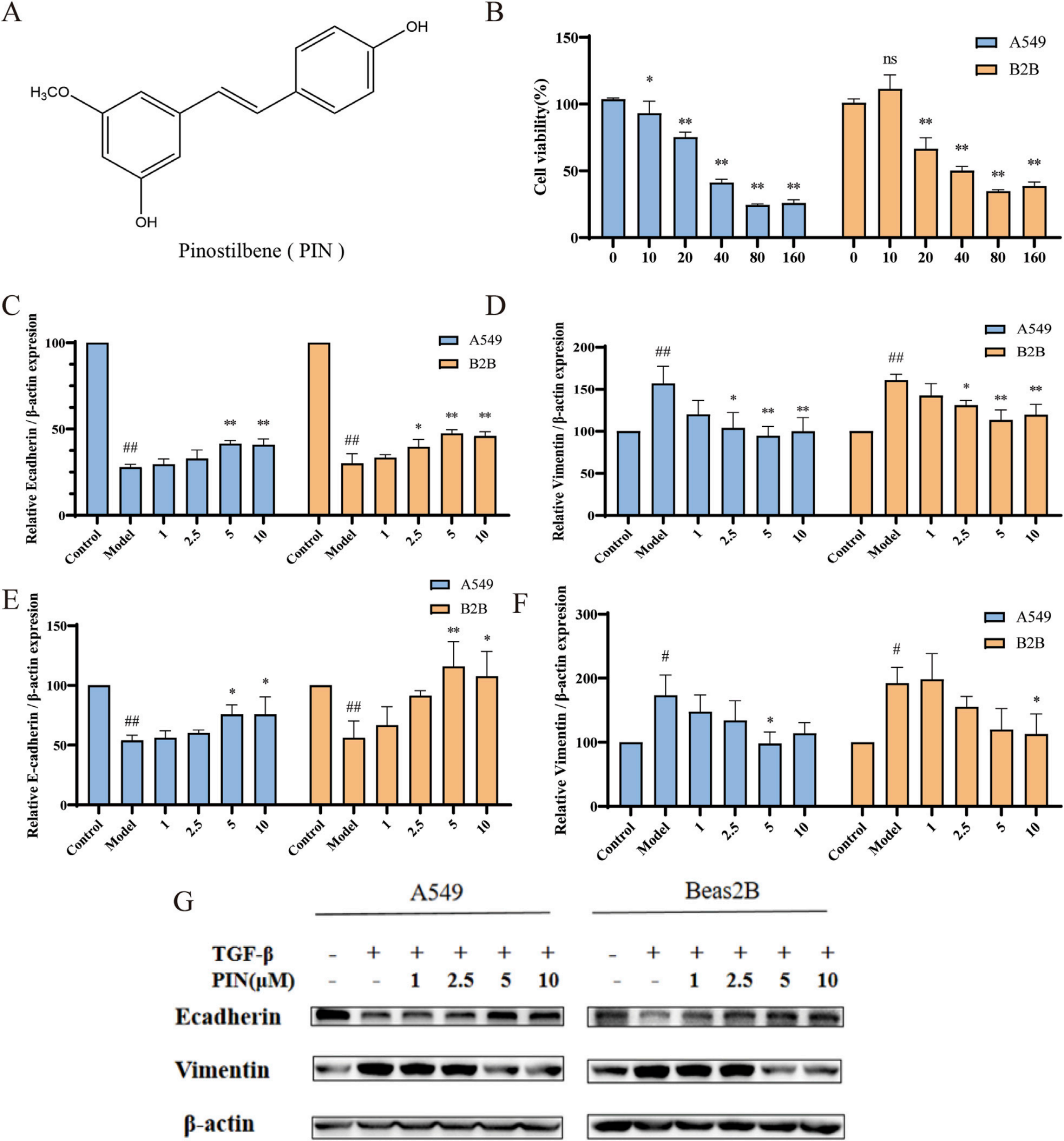
PIN inhibits TGF-β1-induced EMT in A549 and Beas2B cells. **(A)** PIN possesses a stilbene structure. **(B)** The effect of various concentrations of PIN on cell viability after 48 h of incubation with 5 ng/mL TGF-β1 in A549 and Beas2B cells (n = 4). **(C,D)** The impact of a gradient of PIN concentrations on the mRNA expression levels of E-cadherin and Vimentin in TGF-β1-induced A549 and Beas2B cells. **(E–G)** Changes in E-cadherin and Vimentin protein expression levels in TGF-β1-induced A549 and Beas2B cells with varying concentrations of PIN intervention. n = 3; compared with the control group, ^##^ *P* < 0.01, and ^#^ *P* < 0.05; compared with the model group, **P < 0.01, and *P < 0.05; ns indicates no statistically significant difference. Error bars represent the mean ± standard deviation.

A wide range of stilbene compounds has been identified with anti-EMT and anti-fibrotic properties. For example, Resveratrol has been discovered the ability to inhibit EMT across a range of cancer types, including breast cancer (J. Wang et al., 2023), liver cancer (Tong et al., 2024; Song et al., 2021), gastric cancer (Deng et al., 2022), colorectal cancer (Brockmueller et al., 2024) and oral squamous cell carcinoma (Min et al., 2024). Tamoxifen is capable of mitigating the EMT (Lv and Xu, 2022) and fibrosis (Jiang et al., 2024) in endometrial cancer. Pterostilbene has been found to inhibit cellular EMT in diseases such as liver cancer (Song et al., 2019), lung cancer (Peng et al., 2021), gastric cancer (He et al., 2024) and renal fibrosis (Gu et al., 2019). Pinostilbene (as shown in Figure 1, PIN), structurally similar to resveratrol and pterostilbene, is a natural stilbene compound primarily derived from plants of the pine genus (Allen et al., 2018). Previous studies have demonstrated that PIN exerts inhibitory effects in various cancers, including liver and prostate cancer, by suppressing tumor cell proliferation, inducing apoptosis, and inhibiting metastasis through multiple mechanisms (Sun et al., 2016; Hsieh et al., 2018). In terms of antioxidant properties, PIN can effectively scavenge free radicals and mitigate the damage to cells and tissues caused by oxidative stress (Li et al., 2023; Treml et al., 2019). Its anti-inflammatory effects are manifested in the regulation of the secretion and signaling of inflammatory factors, reducing the adverse effects of inflammatory responses on the body (Koh et al., 2022). Additionally, PIN has shown potential in neuroprotection, promoting the survival and functional maintenance of nerve cells (Allen et al., 2018). However, current research on the role of PIN in the context of the lungs is relatively scarce. Given the key role of pulmonary EMT in the development and progression of various pulmonary diseases, including pulmonary fibrosis, further investigation into the effects of PIN on pulmonary EMT is of great significance.

In view of this, delving into the effects of PIN on pulmonary EMT and its underlying mechanisms holds significant scientific importance and potential clinical application value. Results from network pharmacology and molecular docking studies indicate that the PI3K/Akt signaling pathway is the primary pathway through which PIN exerts its anti-pulmonary EMT effects. Preliminary research has shown that the PI3K/Akt signaling pathway plays a crucial regulatory role in various biological processes such as cell proliferation, survival, migration, and EMT, and is closely associated with the onset and progression of pulmonary fibrosis (Fruman et al., 2017). Therefore, this study will focus on this signaling pathway to investigate whether PIN can inhibit pulmonary EMT by modulating the PI3K/Akt signaling pathway. The findings will provide a robust theoretical basis and experimental evidence supporting the potential application of PIN in treating pulmonary fibrosis.

## 2 Materials and methods

### 2.1 Reagents and drugs

TGF-β1 (Cat. Number AF-100-21C-100) was purchased from PeproTech Inc.; Pinostilbene (purity >98%, Cat. Number PCS2302) was ordered from Chengdu purechem-standard co., LTD (Chengdu, China); The antibodies for β-actin (Cat. Number K200058M), E-cadherin (Cat. Number K011355P), Vimentin (Cat. Number K002388P), α-smooth muscle actin (α-SMA,Cat. Number GB111364) were purchased from Solarbio.; The antibodies for phospho-PI3K (Cat. Number AF3241), total PI3K (Cat. Number AF6241), phospho-AKT (Cat. Number AF0016), total AKT (Cat. Number AF6261) were purchased from Affinity biosciences; The activator SC79 (Cat. Number HY-18749) and the inhibitor LY294002 (Cat. Number HY-10108) of the PI3K/Akt pathway were purchased from MedChemExpress (Monmouth Junction, NJ, United States); Bleomycin (BLM) sulfate (Cat. Number HY-17565) were purchased from MedChemExpress (Monmouth Junction, NJ, United States).

### 2.2 Cell culture

A549 and Beas2B cells (Procell, Wuhan, China) were cultured in an incubator at 37 °C in 5% CO_2_ with RPMI-1640 (Gibco, Sigma Aldrich, Søborg, Denmark) containing 10% foetal bovine serum (Gibco, Sigma Aldrich, Denmark) and 1% penicillin-streptomycin (Gibco, Sigma Aldrich, Denmark). Cells were digested with 0.25% trypsin (Gibco, Sigma Aldrich, Denmark) and passaged at 80% confluence.

### 2.3 MTT assay

The effects of PIN on the viability of A549 and Beas2B cells were assessed using the MTT assay. Cells were seeded into a 96-well plate (PerkinElmer, Waltham, MA, United States) at 100 µL per well and incubated for 24 h. PIN was then added to achieve concentrations of 0, 10, 20, 40, 80, and 160 μmol/L, followed by incubation for 48 h. Subsequently, reagents were added according to the MTT kit instructions (Solabio, Beijing, China). The absorbance (OD value) of each well was measured at 490 nm, and cell viability was calculated based on the OD values.

$$\text{Cell survival rate }\left(\%\right)=\left(\text{Experimental group OD value }‐\text{ Blank group OD value}\right)\;/\;\left(\text{Control group OD value }‐\text{ Blank group OD value}\right)\times 100\%$$

### 2.4 RT-qPCR

Total RNA was extracted using SteadyPure Quick RNA Extraction Kit (Accurate Biotechnology (Hunan) Co., Ltd). And then, the RNA was reverse-transcribed into cDNA using Evo M-MLV RT Mix Kit with gDNA Clean for qPCR Ver.2, following the manufacturer’s instructions. The mRNA expression levels were determined by RT-qPCR with SYBR Green Premix Pro Taq HS qPCR Kit and a QuantStudio™ 5 RT-qPCR system (Thermo Fisher, Waltham, MA, United States). The expression levels of E-cadherin and Vimentin were normalized to β-actin. Primer sequences are listed in Table 1.

**TABLE 1:** Primer sequences for use in RT-qPCR.

Target	Primer sequences	
E-cadherin	Forward	5′-GAG​TGC​CAA​CTG​GAC​CAT​TCA​GTA-3′
	Reverse	5′-CAC​AGT​CAC​ACA​CGC​TGA​CCT​CTA-3′
Vimentin	Forward	5′-TGA​CAT​TGA​GAT​TGC​CAC​CTA​CAG-3′
	Reverse	5′-TCA​ACC​GTC​TTA​ATC​AGA​AGT​GTC​C-3′
β-actin	Forward	5′-TGA​CGT​GGA​CAT​CCG​CAA​AG-3′
	Reverse	5′-CTG​GAA​GGT​GGA​CAG​CGA​GG-3′

### 2.5 Western blot assay

For protein extraction and quantification, cells and lung tissue of mice were rinsed with PBS (Solarbio, Beijing, China) and homogenized in RIPA buffer containing 1% phenylmethylsulfonyl fluoride (PMSF) and 1% phosphatase inhibitor (Beyotime, Beijing, China). The homogenates were centrifuged at 13,000 × g for 15 min at 4 °C, and the resulting supernatants were harvested for subsequent Western blot analysis.

The protein concentration was determined using the BCA protein assay kit (EpiZyme, Shanghai, China). Subsequently, 30 µg of protein was resolved by SDS-PAGE and electrotransferred onto a 0.45 µm PVDF membrane (Millipore, MA, United States). Following incubation with High-Efficiency Western Blot Blocking Buffer (Genefist, Shanghai, China), the membrane was subjected to overnight incubation with the primary antibody at 4 °C. After washing, the membrane was further incubated with horseradish peroxidase (HRP)-conjugated Goat Anti-Rabbit IgG (H + L) secondary antibody (1:3000; Solarbio, Beijing, China) for 1.5 h at room temperature. The membranes were examined utilizing an automated chemiluminescence imaging analyzer (Tanon, Shanghai, China) for immunoblot analysis, with densitometric quantification of the gray values accomplished using ImageJ 1.8.0 software.

### 2.6 Adhesion assay

A549 and Beas2B cells were seeded into 6-well plates and incubated for 24 h, after which they were divided into three groups: control group (medium with 1% FBS, no TGF-β1), TGF-β1 group (medium with 1% FBS and 5 ng/mL TGF-β1), and compound group (medium with 1% FBS, 5 ng/mL TGF-β1, and 5 µM PIN). Cells were then incubated for 48 h. Fibronectin (FN, 0.2 mg/mL, Solarbio, Beijing, China) was diluted with PBS to 100 µL per well, added to 96-well plates, dried for 60 min, and stored at 4 °C overnight. Cells were transferred from the 6-well plates to the 96-well plates and incubated for 45 min for staining. Cells were fixed with 4% formaldehyde for 10 min, permeabilized with 0.5% Triton X-100 for 5 min, and stained with 10 μg/mL Hoechst 33342. Fluorescence images were captured using a microscope (OLYMPUS, Japan) and analyzed using ImageJ to quantify cell adhesion following treatment with different compounds.

### 2.7 Scratch assay

A549 and Beas2B cells were introduced into 6-well plates and maintained in a serum-free medium for 24 h. To create an *in vitro* scratch model, a 200 µL sterilized pipette tip was used to make vertical scratches along premarked lines, ensuring uniform scratch intensity and width across all wells. Following scratching, the wells were rinsed with PBS until no cell debris remained in the scratched areas, and images were taken using an inverted microscope. In group settings, according to the determination of adhesion, drug-containing serum-free medium was added to the designated wells and incubated for 48 h before imaging. The scratch area was quantified at various time points using ImageJ software to determine cell migration.

$$\text{Migration rate }\left(\%\right)=\left(\text{Scratch distance at }0h\;‐\text{ Scratch distance at }48h\right)\;/\text{ Scratch distance at }0h\times 100\%$$

### 2.8 Animal experiments

The animal experiments detailed in this study were approved by the Animal Ethics Committee of Shandong University of Traditional Chinese Medicine (approval no. SDUTCM20241108003). SPF-grade male C57BL/6J N mice (20 ± 2 g) were acquired from Beijing Viton Lever Laboratory Animal Technology Co, Ltd. (Beijing, China; animal certificate number of SYXK (Lu) 20220009). The animal housing facility was kept at 22.9 °C, with 46.6% relative humidity and a 12 h/12 h light/dark cycle.

For experimentation, 48 mice were randomly assigned into the following six groups (n = 8): Control, Model, pirfenidone group (PFD), BLM +30 mg/kg PIN groups (H), BLM +15 mg/kg PIN groups (L) and the combination group of high-dose PIN and pirfenidone (PIN + PFD), with the aim of evaluating the synergistic effects of PIN and pirfenidone *in vivo*. Bleomycin sulfate solution (2.5 mg/kg) was administered intratracheally to establish the idiopathic pulmonary fibrosis (IPF) mouse model, while the control group received an intratracheal injection of saline. After 7 d, the H group and the L group were given PIN by gavage at the indicated doses (30 and 15 mg/kg) daily, the PFD group was given 300 mg/kg pirfenidone by gavage and the PIN + PFD group was given both 30 mg/kg of PIN and 300 mg/kg of pirfenidone simultaneously. Seven days after modeling, Group H and Group L were respectively given intragastric administration of PIN at the prescribed doses (30 and 15 mg/kg) daily. Rats in the PFD group were given pirfenidone 300 mg/kg by gavage. The PIN + PFD group was simultaneously given a PIN dose of 30 mg/kg and a pirfenidone dose of 300 mg/kg. After continuous administration for 21 days, the mice were sacrificed. The left lungs were fixed in 10% formalin at 26 °C and prepared for hematoxylin-eosin (H&E) and Masson staining. Concurrently, the right lungs were snap-frozen in liquid nitrogen and stored at −80 °C for western blotting and RT-qPCR analyses.

### 2.9 Histological analyses

Record the body weight and the wet weight of the lung tissue of the mice. Calculate the lung coefficient using the formula: Pulmonary index = (wet lung weight/body weight) × 100%. Lung tissues were fixed in 4% paraformaldehyde (pH 7.4), embedded in conventional paraffin, sectioned, and then selected for H&E and Masson staining. Histopathological and fibrotic alterations in the lung tissues were examined using a light microscope (Olympus, Japan).

### 2.10 Bioinformatics analysis

Disease genes were integrated by searching EMT and IPF in the Genecards and OMIM databases and taking the union for consolidation. Drug targets were identified through the PharmMapper website. The intersection of disease genes and drug genes was obtained using Venny 2.1.0. The resulting drug-disease targets were imported into the STRING database to construct a protein-protein interaction (PPI) network, which was further visualized using Cytoscape 3.10.0 software. GO and KEGG pathway enrichment analysis for PIN-EMT targets were performed in the DAVID database. In Excel, significant enrichment entries were processed according to Term, Gene ratio, P-value, and Count, and the data were imported into the bioinformatics online platform (www.bioinformatics.com.cn) for visualization analysis, presenting the data in the form of bubble charts.

Molecular docking was performed between PIN and its target proteins PI3K and Akt. The three-dimensional structure of PIN in SDF format was downloaded from the PubChem website and converted to mol.2 format using Open Babel software. The crystal structures of the target proteins were retrieved from the RCSB Protein Data Bank (PDB) at https://www.rcsb.org, and water molecules and ligands were removed using PyMOL 2.6 software, which can be accessed at http://www.pymol.org. The structures were then imported into AutoDocktools 4.2.6 software, available at https://autodock.scripps.edu/, to convert them into PDBQT format for molecular docking, which generated binding energies. The results of the docking were visualized using PyMOL 2.6 software.

### 2.11 Statistical analysis

All statistical analysis was performed with GraphPad Prism software version 9.0. Data are expressed as mean ± standard error of the mean (SEM). Comparison between groups was analyzed by one-way analysis of variance (ANOVA) or Student’s t-test. A value of p < 0.05 (compared with the control group, ##P < 0.01, and #P < 0.05; compared with the model group, **P < 0.01, and *P < 0.05; ns indicates no statistically significant difference) was considered to indicate statistical significance.

## 3 Results

### 3.1 PIN inhibits TGF-β1-induced cell EMT

#### 3.1.1 PIN inhibits TGF-β1-driven EMT expression

The chemical structure of PIN is depicted in Figure 1A. Employing A549 and Beas2B cell lines, we investigated the effects of diverse concentrations of PIN on cellular viability, with results depicted in Figure 1B. MTT assays determined the IC10 values for A549 and Beas2B cells to be 9 μM and 15.15 μM, respectively. At the same time, we investigated the anti-EMT effect with different concentrations of PIN, and found that the anti-EMT effect was more significant with the increase of PIN dose, and considering the cytotoxicity, we chose the subsequent up-concentration of 5 μM and 10 μM overexposure for the subsequent experiments (Supplementary Figure S1). EMT induction was confirmed in both cell lines following incubation with 5 ng/mL TGF-β1 for 48 h, as demonstrated by qRT-PCR and western blot analyses. Compared to the control group, the TGF-β1-treated group (Model) exhibited reduced mRNA and protein expression of E-cadherin and increased expression of Vimentin. Treatment with PIN at varying concentrations reversed these effects, increasing E-cadherin levels and decreasing Vimentin levels in a concentration-dependent manner, as presented in Figures 1C–F. Notably, no significant distinction in the inhibition of EMT was found between the 5 μM and 10 μM concentrations of PIN. Consequently, a concentration of 5 μM PIN was chosen for subsequent experimental investigations.

#### 3.1.2 PIN inhibits TGF-β-induced cell adhesion and migration

**FIGURE 2:**
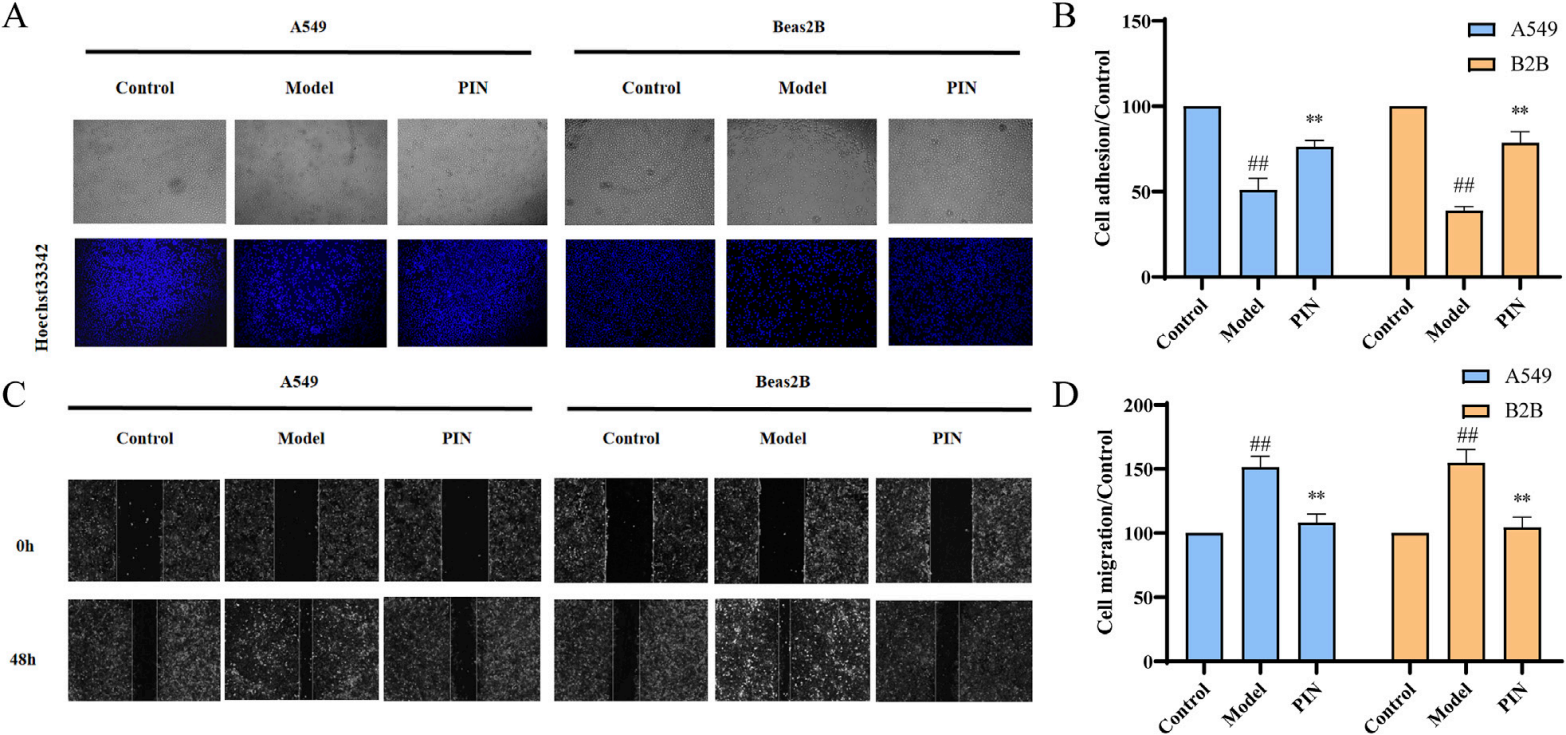
Adhesion and scratch assays of TGF-β1-induced A549 and Beas2B cells treated with 5 μM PIN. **(A)** Representative stained adhesion images of A549 and Beas2B cells after 48 h. **(B)** Adhesion of A549 and Beas2B cells after 48 h. **(C)** Migration of A549 and Beas2B cells after 48 h. **(D)** Migration of A549 and Beas2B cells after 48 h. Mean ± standard deviation; n = 3; compared to the control group, ^##^ *P* < 0.01; compared to the model group, **P < 0.01. Magnification: 100 times.

The EMT process significantly enhances the invasiveness and migratory potential of cells, thereby severely impacting the therapeutic outcomes and patient prognosis in fibrotic tissues and cancer (Liaghat et al., 2024; Yuan et al., 2024; Martínez-Espinosa et al., 2024). To investigate the behavioral changes in cells during EMT and potential intervention effects, we performed a cell adhesion assay to measure alterations in cell adhesion to the extracellular matrix and a scratch assay to evaluate cell migration. Results from the cell adhesion assay (Figures 2A,B) showed a significant reduction in the number of adherent cells in the Model group compared to the control group (p < 0.01). Treatment with PIN effectively reversed this reduction (p < 0.01). Similarly, scratch assay results (Figures 2C,D) demonstrated a marked decrease in scratch distance in the Model group, indicative of increased cell migration ability (p < 0.01). PIN treatment partially mitigated this effect, reducing cell migration (p < 0.01).

### 3.2 PIN inhibits bleomycin-induced EMT in C57BL/6J male mice

**FIGURE 3:**
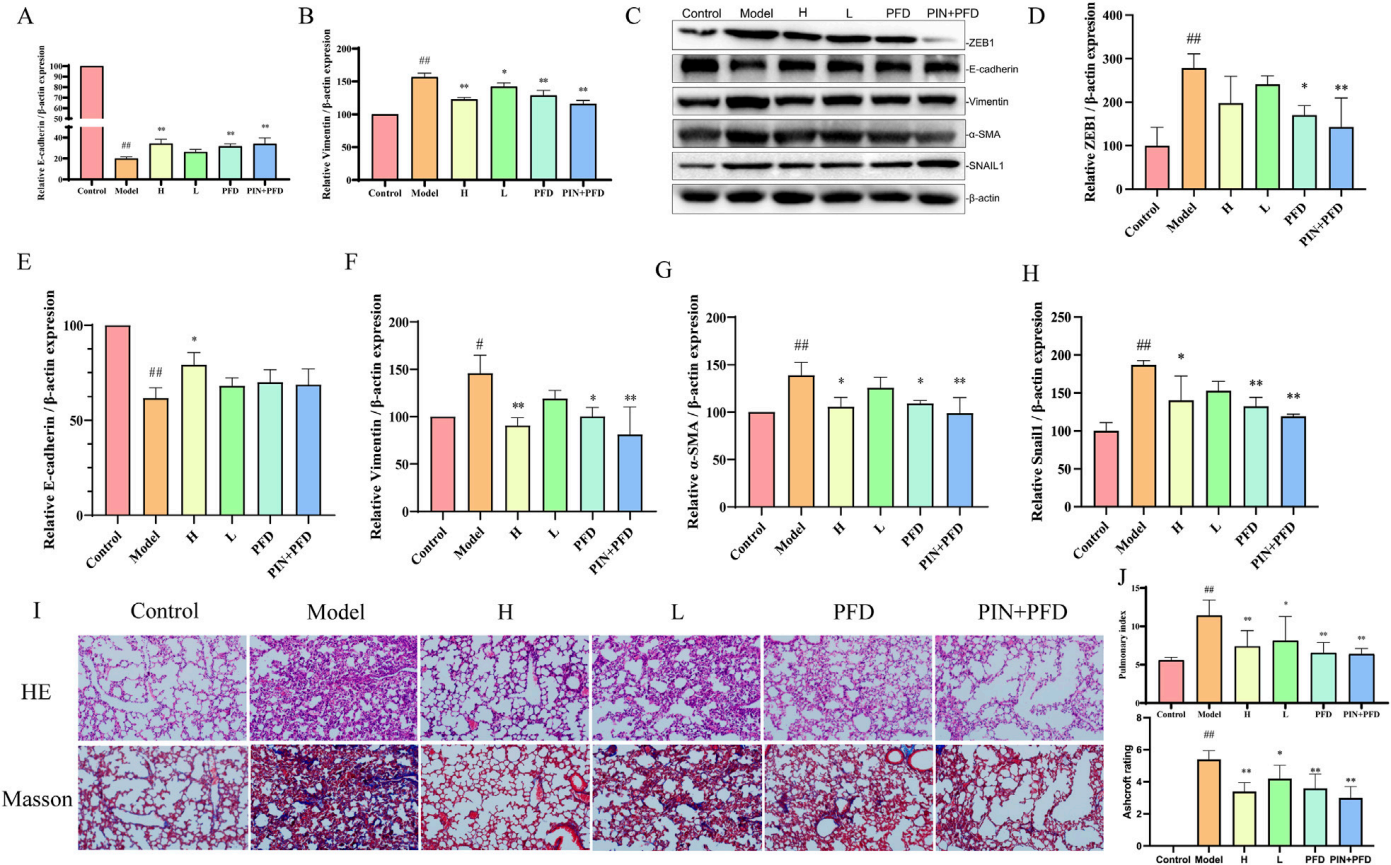
Inhibition of Bleomycin-Induced EMT by PIN in C57BL/6J Male Mice. **(A,B)** The suppressive effect of PIN on the expression of genes associated with bleomycin-induced pulmonary fibrosis. **(C–H)** The suppressive effect of PIN on the expression of proteins associated with bleomycin-induced pulmonary fibrosis. **(I)** Representative images of H&E (top panel) and Masson’s trichrome (bottom panel) staining of mouse lung tissue on day 28. Magnification: 200 times. **(J)** Pulmonary index and Ashcroft scoring in mice. Data are presented as mean ± standard deviation; n = 3; statistical significance compared to the control group is denoted as ^##^ *P* < 0.01, and ^#^ *P* < 0.05; compared to the model group as **P < 0.01, and *P < 0.05; ns signifies no statistically significant difference. H: BLM +30 mg/kg PIN groups; L: BLM +15 mg/kg PIN groups; PFD: The pirfenidone group; PIN + PFD: The combination group of high-dose PIN and pirfenidone.

Bleomycin serves as a well-established and potent agent for generating pulmonary fibrosis models (Sangaraju et al., 2024; Li K. et al., 2024). Models induced by bleomycin closely mimic the sequence of pathophysiological alterations observed in human lung tissue during the course of pulmonary fibrosis, allowing for an effective assessment of PIN’s impact on pulmonary epithelial-mesenchymal transition within this particular pathological context (Mohammed et al., 2024). After 21 days of PIN treatment, the Pulmonary Index in the bleomycin-induced model group was significantly higher than in the control group (p < 0.01, Figure 3A). High-dose PIN treatment significantly reduced the Pulmonary Index (p < 0.01), with an even greater reduction observed in the PFD group (p < 0.01) and the PIN + PFD group (p < 0.01).

RT-qPCR (Figures 3B,C) revealed that the model group exhibited significant downregulation of E-cadherin (p < 0.01) and upregulation of Vimentin gene expression (p < 0.01) compared to the control group. These EMT-associated gene expression changes were significantly reversed by high-dose PIN, pirfenidone, and their combination treatments. Protein analysis (Figures 3D–G) showed a decrease in E-cadherin levels (p < 0.01) and an increase in Vimentin (p < 0.05) and α-SMA (p < 0.01) in the model group relative to controls, which were effectively mitigated by the treatment groups. After Pin treatment, the expression levels of EMT-related transcription factors ZEB1 and Snail1 were significantly reduced, which proves that PIN can reduce the occurrence of EMT by inhibiting the expression of related transcription factors.

H&E staining revealed that control animals exhibited normal lung tissue architecture, while bleomycin-treated animals displayed distorted morphology characterized by inflammatory cell infiltration, thickened alveolar septa, alveolar edema, and collapsed alveolar spaces with inflammatory exudate. The high-dose PIN, pirfenidone, and combination therapy groups demonstrated marked improvement in these pathological features. Masson’s trichrome staining further indicated minimal collagen deposition in control animals but extensive fibrosis and collagen fiber accumulation in the bleomycin group. Notably, the high-dose PIN, pirfenidone, and combination therapy groups significantly reduced collagen deposition compared to the bleomycin group. The degree of fibrosis in several boxes of lung tissue was scored using the Ashcroft score. The results showed that after PIN treatment, the degree of fibrosis was significantly reduce.

### 3.3 Network pharmacology of PIN

#### 3.3.1 Core Targets of PIN in EMT treatment through PPI Network Analysis

**FIGURE 4:**
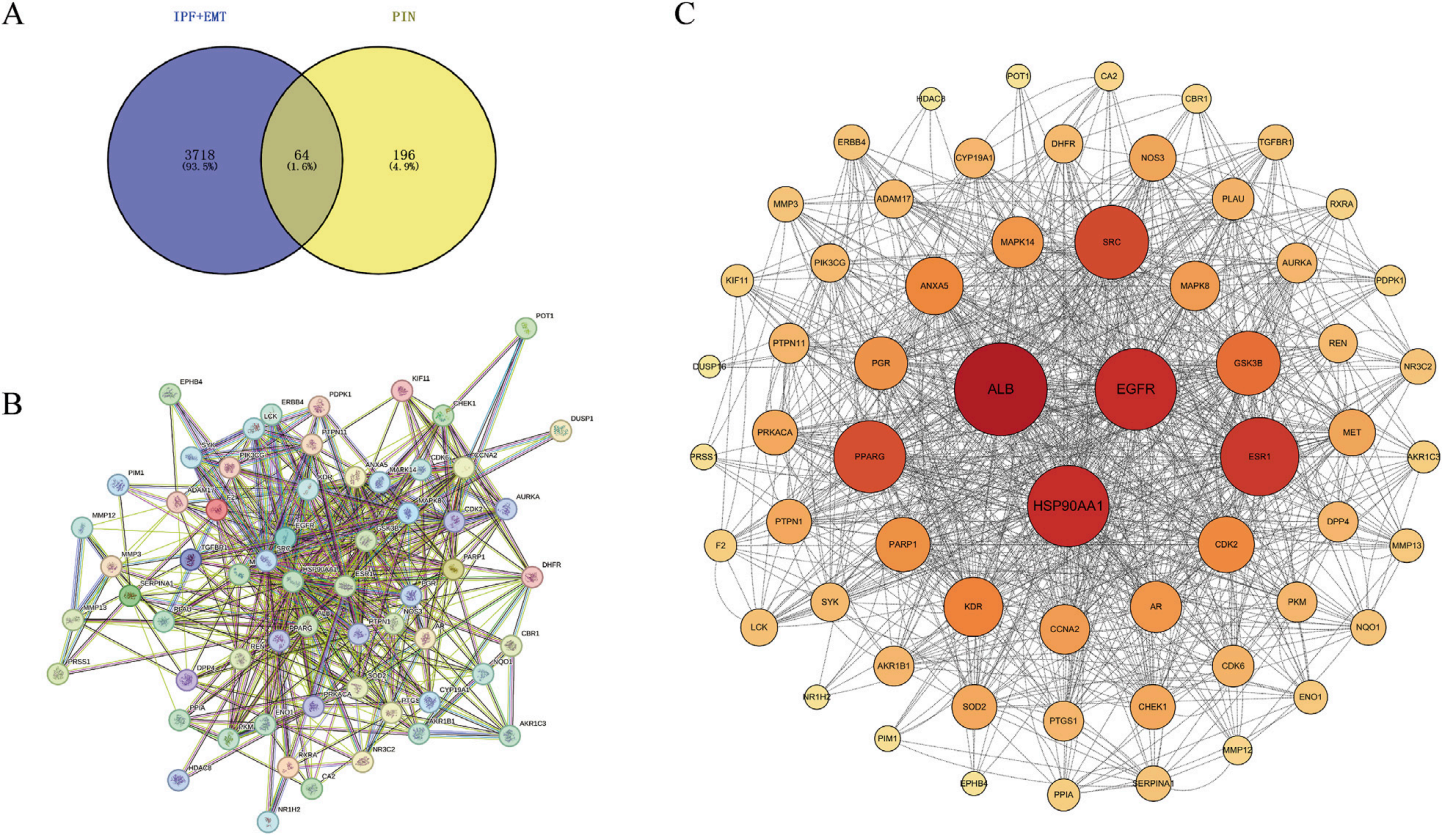
Identification of Core Targets of PIN Against EMT through PPI Network Analysis. **(A)** PIN-related targets associated with EMT and IPF were imported into Venny 2.1.0 to construct a Venn diagram, revealing 64 drug-disease intersecting targets. **(B)** The PPI network was constructed using the STRING database, and **(C)** visualized with Cytoscape 3.10.0. Nodes represent targets, with the size and color intensity of each node positively correlating with its degree value. Edges connecting the nodes signify the interactions among the targets.

Genes associated with disease were identified by querying the Genecards and OMIM databases for EMT and IPF. After integrating the search results through a union operation, a total of 3,718 unique genes were obtained. Additionally, drug targets were determined using the PharmMapper website, resulting in a total of 196 targets. Utilizing Venny 2.1.0, we found the intersection of disease and drug genes, identifying a total of 64 common targets (Figure 4A). To delineate the key targets of PIN in EMT, these 64 drug-disease targets were imported into the String database, generating a protein-protein interaction network (Figure 4B), which was subsequently visualized with Cytoscape 3.10.0 software (Figure 4C). The findings highlighted ALB, EGFR, and HSP90AA1 as central nodes within the PPI network, with the highest degree values indicating their significance (Table 2).

**TABLE 2:** Summary of the 20 targets in the PPI network diagram for the treatment of EMT and IPF with PIN.

Number	Target name	Degree
1	ALB	92
2	HSP90AA1	78
3	EGFR	78
4	ESR1	74
5	SRC	68
6	PPARG	66
7	GSK3B	56
8	KDR	50
9	ANXA5	48
10	CDK2	46
11	PARP1	44
12	PGR	42
13	AR	40
14	MAPK14	40
15	CCNA2	38
16	MAPK8	38
17	MET	34
18	NOS3	34
19	PRKACA	32
20	CHEK1	32

#### 3.3.2 GO and KEGG pathway enrichment analysis

**FIGURE 5:**
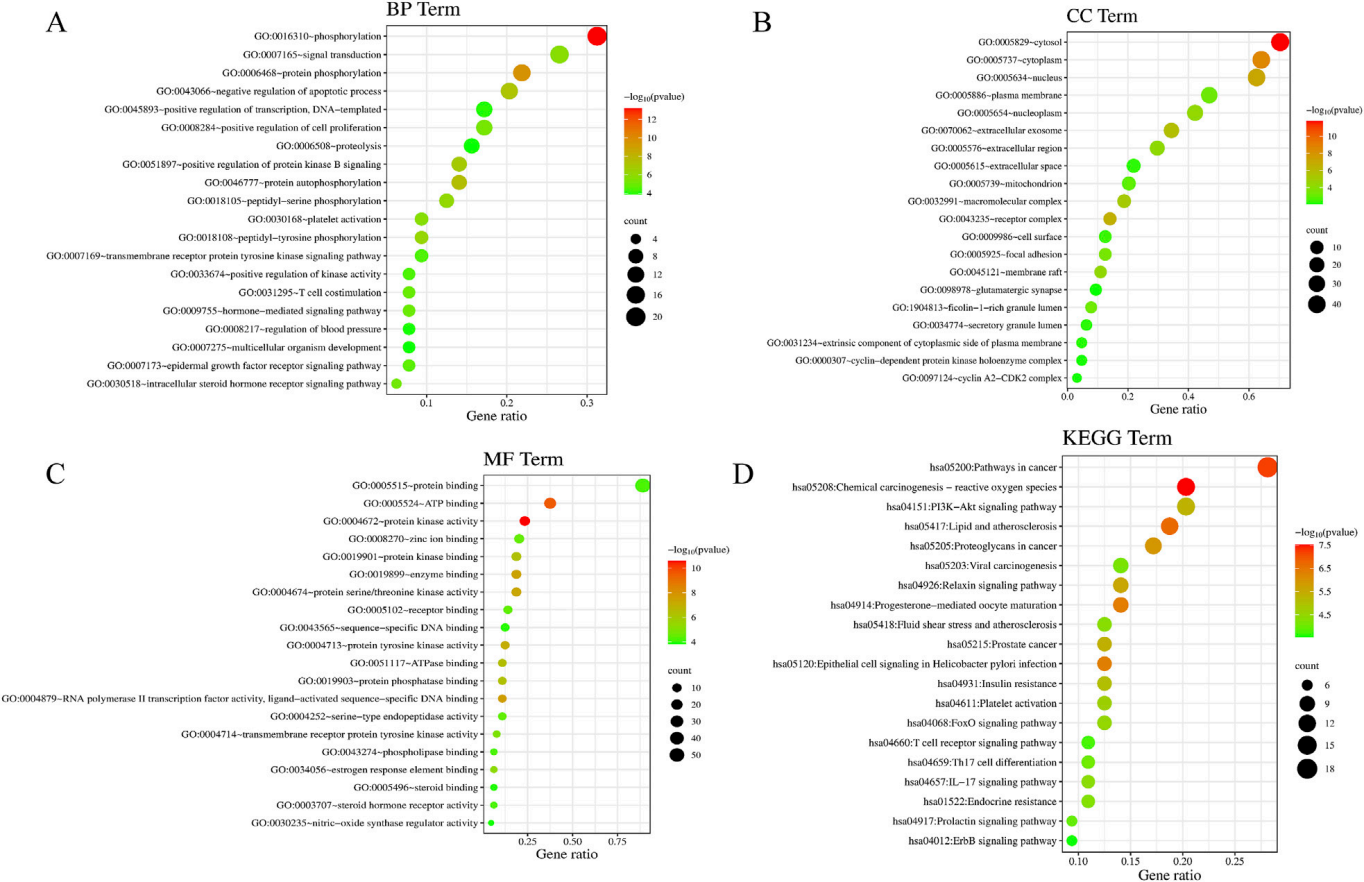
Enrichment Analysis of GO and KEGG Pathways in PIN Treatment of EMT. The GO enrichment analysis includes BP **(A)**, CC **(B)**, and MF **(C)**, depicted as bubble charts. KEGG pathway enrichment **(D)** highlights the correlation with the PI3K/Akt signaling pathway in PIN treatment. The x-axis and y-axis indicate the gene ratio and enrichment clusters, respectively, with bubble coloration and size representing p-values and gene counts, respectively.

GO and KEGG pathway enrichment analyses were performed on the 64 PIN-EMT targets using the DAVID database. A total of 322 Gene Ontology (GO) terms were significantly enriched, including 208 biological processes (BP), 36 cellular components (CC), and 78 molecular functions (MF). The top 20 data points were visualized on a bioinformatics online platform, with the gene ratio plotted on the x-axis and sorted by p-value (Figure 5). The primary BP clusters included phosphorylation, signal transduction, protein phosphorylation, negative regulation of apoptosis, positive regulation of transcription from a DNA template, positive regulation of cell proliferation, and proteolysis (Figure 5A). The main CC clusters comprised the cytosol, cytoplasm, nucleus, plasma membrane, nucleoplasm, extracellular exosome, and extracellular region (Figure 5B). The predominant MF clusters were protein binding, ATP binding, protein kinase activity, zinc ion binding, and enzyme binding (Figure 5C). These results suggest that PIN may influence EMT by modulating protein phosphorylation, regulating signal transduction pathways, activating transcription factors, and altering interactions between the cell membrane and cytoplasm. Additionally, KEGG pathway enrichment analysis identified 100 enriched KEGG terms, with the top 20 presented in a bubble chart (Figure 5D). Notably, the PI3K/Akt signaling pathway emerged as a key pathway regulated by PIN in the context of EMT, as shown in Table 3.

**TABLE 3:** Summary of the top 20 targets for the Intersecting Targets between PIN, EMT, and IPF in a PPI network.

ID	Description	Gene ratio	*p*-valμe	Count
hsa05200	Pathways in cancer	18/64	6.20E-08	18
hsa05208	Chemical carcinogenesis - reactive oxygen species	13/64	2.97E-08	13
hsa04151	PI3K-Akt signaling pathway	13/64	4.95E-06	13
hsa05417	Lipid and atherosclerosis	12/64	2.01E-07	12
hsa05205	Proteoglycans in cancer	11/64	1.19E-06	11
hsa05203	Viral carcinogenesis	9/64	7.38E-05	9
hsa04926	Relaxin signaling pathway	9/64	2.60E-06	9
hsa04914	Progesterone-mediated oocyte maturation	9/64	4.33E-07	9
hsa05418	Fluid shear stress and atherosclerosis	8/64	4.47E-05	8
hsa05215	Prostate cancer	8/64	4.22E-06	8
hsa05120	Epithelial cell signaling in *Helicobacter pylori* infection	8/64	4.56E-07	8
hsa04931	Insulin resistance	8/64	8.64E-06	8
hsa04611	Platelet activation	8/64	2.14E-05	8
hsa04068	FoxO signaling pathway	8/64	3.06E-05	8
hsa04660	T cell receptor signaling pathway	7/64	1.77E-04	7
hsa04659	Th17 cell differentiation	7/64	9.47E-05	7
hsa04657	IL-17 signaling pathway	7/64	4.34E-05	7
hsa01522	Endocrine resistance	7/64	5.50E-05	7
hsa04917	Prolactin signaling pathway	6/64	1.15E-04	6
hsa04012	ErbB signaling pathway	6/64	2.89E-04	6

#### 3.3.3 Molecular docking

**FIGURE 6:**
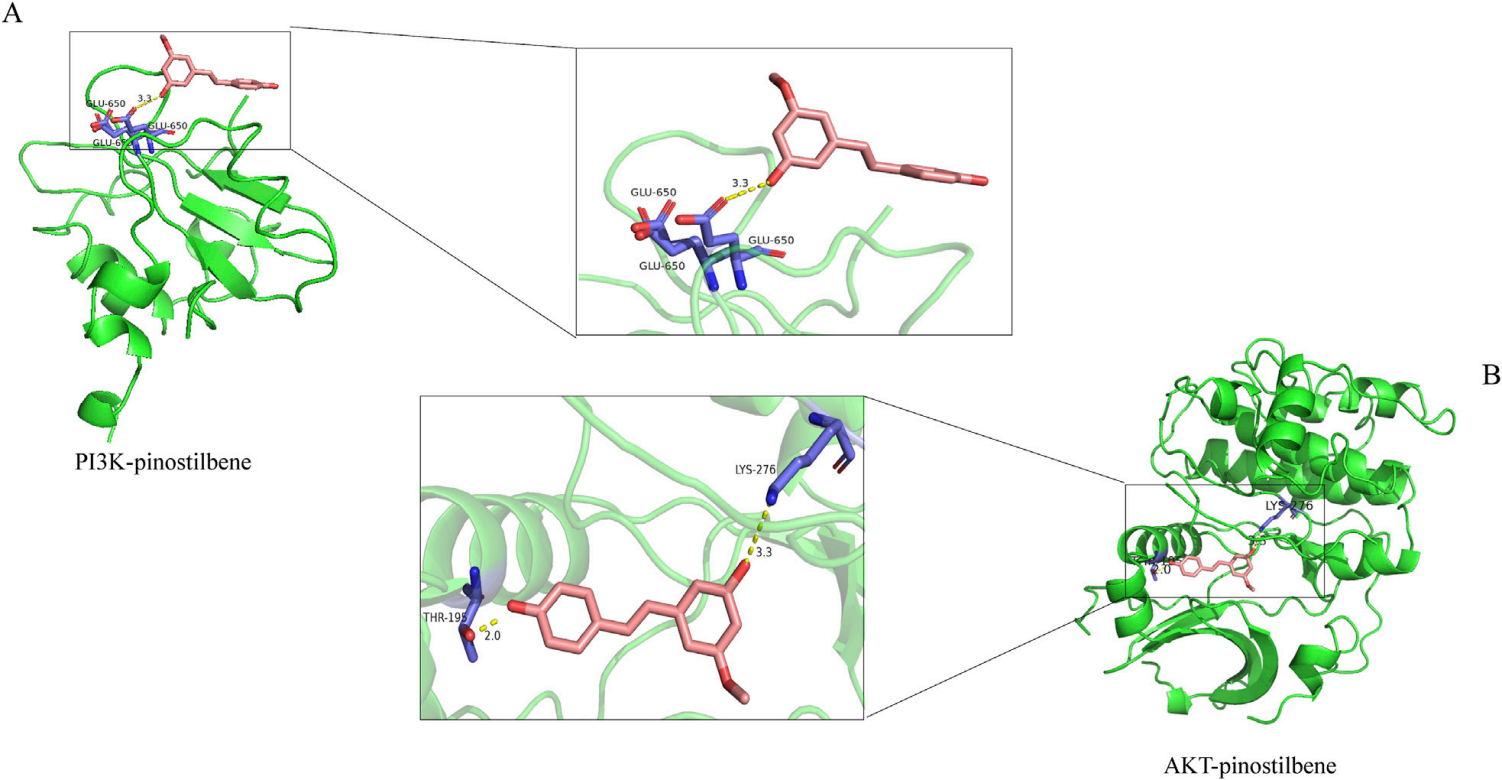
Molecular Docking of PIN with Target Proteins. AutoDocktools 4.2.6 was utilized to perform the docking of PIN with PI3K **(A)** and AKT **(B)**, and the outcomes were visualized with PyMOL 2.6.

Molecular docking technology, due to its ability to accurately simulate the interaction details between small molecules and biomacromolecules, and rapidly predict their binding modes and affinities, is widely applied in various fields such as drug discovery, drug design, and elucidation of mechanisms of action (Nguyen and Ondrus, 2024). In this study, molecular docking analysis was employed to investigate the binding characteristics of PIN with EMT targets. To further substantiate the relevance of PIN with the PI3K/Akt signaling pathway, docking experiments were conducted between PIN and key regulatory proteins within this pathway: PI3K (PDB ID: 5aul) and AKT (PDB ID: 3mv5) (Figure 6). The docking results indicated that the binding energy of PIN with 5aul was −8.4 kcal/mol, and with 3mv5 it was −7.3 kcal/mol, both of which are less than −5.0 kcal/mol (Table 4). Generally, a binding energy below −5.0 kcal/mol is considered to represent a strong interaction between a receptor and a ligand, with lower energies signifying stronger binding affinities (Jia et al., 2024). These findings indicate that PIN exhibits strong binding affinity for both PI3K and AKT, potentially modulating their activation or disrupting their interactions with other molecular entities.

**TABLE 4:** Molecular docking of PIN with PI3K and AKT.

Target name	PDB ID	Binding energy (kcal/mol)
PI3K	5aul	−8.4
AKT	3mv5	−7.3

### 3.4 PIN targets the PI3K/Akt pathway to inhibit EMT

#### 3.4.1 PIN suppresses the activation of the PI3K/Akt signaling pathway in cells

**FIGURE 7:**
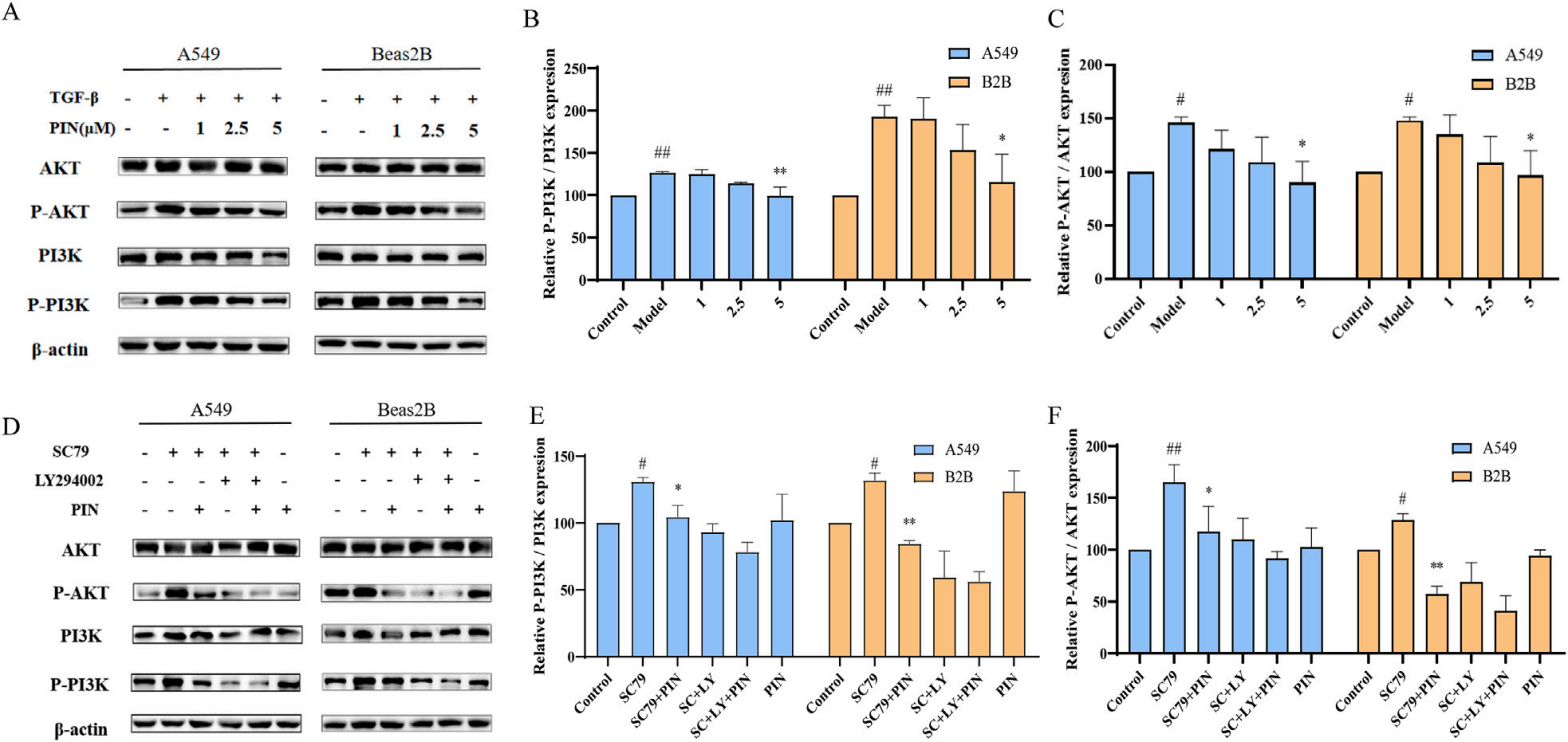
Inhibition of PI3K/Akt signaling pathway activation in A549 and Beas2B cells by PIN. **(A–C)** A549 and Beas2B cells were treated with TGF-β1 (5 ng/mL) in the presence of varying concentrations of PIN (1, 2.5, and 5 μM) for a duration of 48 h. Subsequently, cell lysates were harvested and subjected to western blot analysis to evaluate the expression levels of PI3K, p-PI3K, Akt, and p-Akt. Mean ± standard deviation; n = 3; compared to the control group, ^##^ *P* < 0.01, and ^#^ *P* < 0.05; compared to the model group, **P < 0.01, and *P < 0.05; ns indicates no statistically significant difference. **(D–F)** Examination of the Impact of SC79 and LY294002 on the PI3K/Akt Pathway in A549 and Beas2B Cells. Cells were exposed to SC79 (20 μM) and LY294002 (10 μM) along with 5 μM PIN for a period of 48 h. Following this treatment, cell lysates were obtained and analyzed for the expression of PI3K, p-PI3K, Akt, and p-Akt using western blotting techniques. Mean ± standard deviation; n = 3; compared to the control group, ^##^ *P* < 0.01, and ^#^ *P* < 0.05; compared to the SC79 group, **P < 0.01, and *P < 0.05; ns indicates no statistically significant difference.

The PI3K/Akt signaling pathway has been shown to be a key player in TGF-β1-induced EMT (Chen et al., 2017; Yeh et al., 2016; Wardhani et al., 2021). Based on the results from network pharmacology and molecular docking, the mechanism by which PIN inhibits EMT may operate through this pathway. Following our initial findings, we proceeded to evaluate the activation levels of the pivotal proteins PI3K and Akt during the EMT process utilizing western blot analysis. As shown in Figures 7A,B, the levels of phosphorylated PI3K and Akt in A549 and Beas2B cells treated with TGF-β1 were significantly higher than in the control group. PIN treatment significantly inhibited the activation of p-PI3K and p-Akt in a dose-dependent manner. To confirm the specific targets of PIN within the PI3K/Akt pathway, we used the PI3K/Akt pathway activator SC79 and inhibitor LY294002 (Yin et al., 2020). As depicted in Figures 7C,D, SC79 significantly activated p-PI3K and p-Akt, and PIN effectively reversed these effects. In comparison with the groups treated with SC79 and LY294002, the co-treatment with PIN did not further increase the expression of phosphorylated proteins.

#### 3.4.2 PIN suppresses the activation of the PI3K/Akt signaling pathway in mice

**FIGURE 8:**
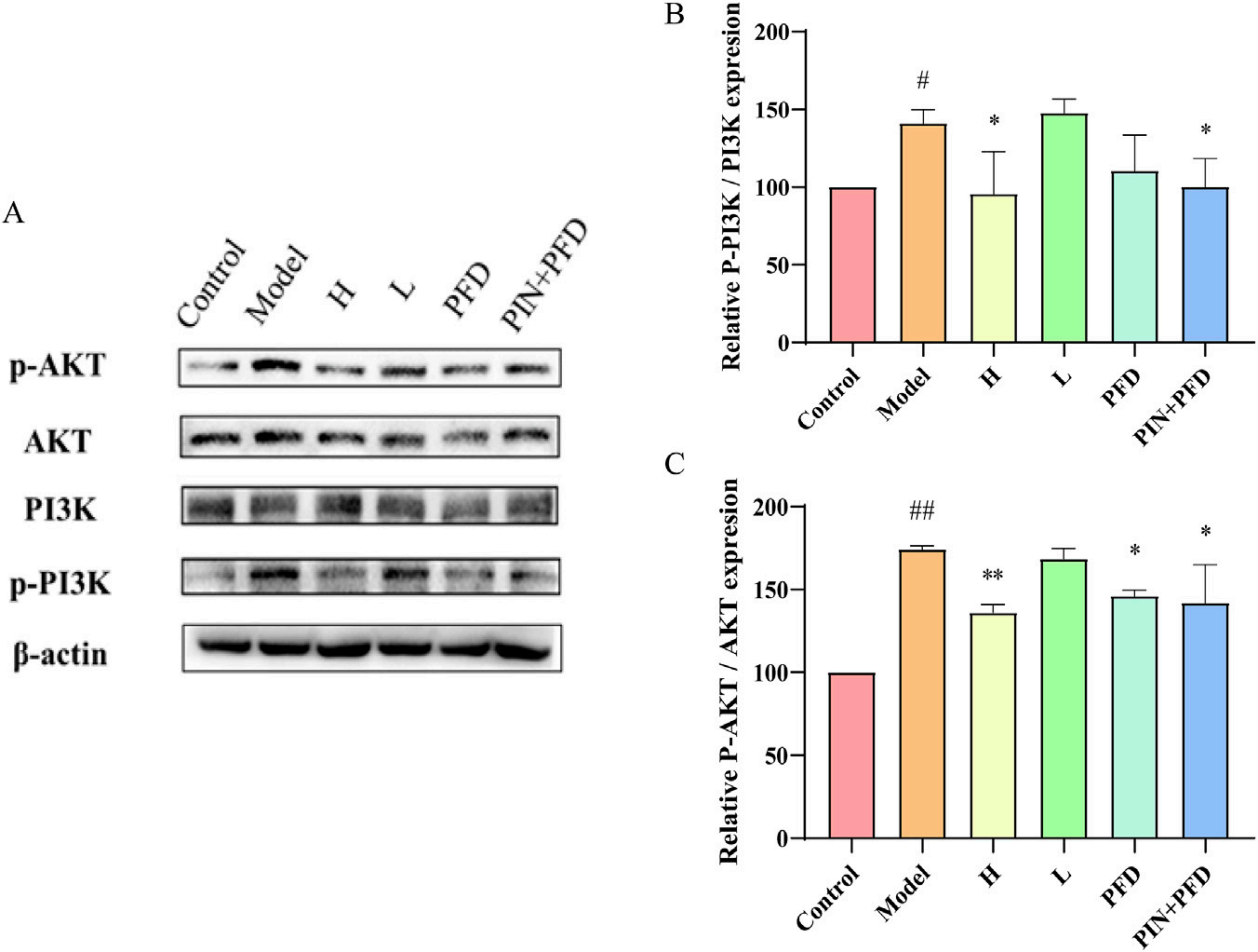
PIN suppresses the activation of the PI3K/Akt signaling pathway induced by bleomycin in C57BL/6J male mice. **(A)** The relationship between PIN and the PI3K/Akt pathway in bleomycin-induced mice was investigated through western blot analysis of PI3K, p-PI3K, Akt, and p-Akt. **(B)** The relative expression levels of p-Akt protein normalized to Akt. **(C)** The relative expression levels of p-PI3K protein normalized to PI3K. Mean ± standard deviation; n = 3; compared to the control group, ^##^ *P* < 0.01, and ^#^ *P* < 0.05; compared to the model group, **P < 0.01, and *P < 0.05; ns indicates no statistically significant difference. H: BLM +30 mg/kg PIN groups; L: BLM+15 mg/kg PIN groups; PFD: The pirfenidone group; PIN + PFD: The combination group of high-dose PIN and pirfenidone.

Previous studies have confirmed that PIN can inhibit bleomycin-induced pulmonary fibrosis in C57BL/6J male mice. To further explore the relationship between PIN and the PI3K/Akt pathway in bleomycin-induced mice, we assessed the protein expression levels of PI3K, p-PI3K, Akt, and p-Akt in lung tissues via western blot analysis, as illustrated in Figure 8. The findings revealed that, relative to the control group, the model group displayed elevated protein levels of p-PI3K (P < 0.05) and p-Akt (p < 0.01). Conversely, the high-dose PIN group, the pirfenidone group, and the combination therapy group all exhibited a decrease in p-PI3K and p-Akt protein expression relative to the model group.

## 4 Discussion

TGF-β1 is a versatile cytokine secreted by diverse cell types, encompassing immune cells, fibroblasts, and epithelial cells. It holds a critical position in a myriad of physiological and pathological processes, including cellular proliferation, differentiation, apoptosis, as well as the synthesis and deposition of the extracellular matrix (ECM) (Li Z. R. et al., 2024). Within the framework of EMT, TGF-β1 is capable of triggering a cascade of intracellular signaling pathways, downregulating the expression of E-cadherin, and upregulating the expression of mesenchymal marker genes, such as Vimentin and α-SMA (Yazaki et al., 2021). In our preceding studies, we have determined the optimal conditions for TGF-β1 to induce cellular EMT (Xu et al., 2022). In this study, we utilized a concentration of 5 ng/mL TGF-β1 to trigger EMT in A549 and Beas2B cells. We observed that TGF-β1 notably changed the morphology of these cells, transitioning from a characteristic epithelial shape to a form reminiscent of mesenchymal cells. Simultaneously, there was a significant decrease in the expression of the epithelial marker E-cadherin, accompanied by a substantial increase in the expression of the mesenchymal marker Vimentin.

Bleomycin-induced pulmonary fibrosis is intricately linked pulmonary EMT. On one hand, bleomycin induces lung tissue injury by generating reactive oxygen species (ROS), leading to the release of TGF-β1, which in turn activates a series of intracellular signaling pathways closely associated with EMT, initiating a cascade of processes including EMT for repair and remodeling (Manavi et al., 2024; Hirano and Takefuji, 2024). On the other hand, during the process of bleomycin-induced pulmonary fibrosis, the morphological and functional changes that occur in pulmonary epithelial cells are consistent with the characteristics of epithelial-to-mesenchymal transition (Zhao et al., 2015; Guo et al., 2015; Li et al., 2017). In previous experiments, we selected bleomycin-induced pulmonary fibrosis in mice to reflect EMT markers (Wang et al., 2021). In this study, we induced pulmonary fibrosis in mice using 2.5 ng/mL bleomycin and initiated therapeutic administration 1 week after establishing the model, which was after the histological evidence of fibrosis had appeared (no earlier than days 7–10) (Jenkins et al., 2017). H&E staining and Masson’s trichrome staining of mouse lung tissue sections demonstrated that the bleomycin group exhibited increased fibrosis, with destruction of the alveolar structure. Additionally, the expression trends of genes and proteins associated with EMT were found to be in agreement with the outcomes of *in vitro* cellular studies.

Among numerous potential therapeutic approaches, natural compounds have attracted significant interest due to their reduced toxicity and distinct modes of action. For example, the active component cupressus funebris total saponins, derived from plants in the Cupressaceae family, have demonstrated *in vitro* antitumor activity, likely through influencing cell cycle progression and promoting apoptosis, thereby inhibiting the proliferation of tumor cells (Zhang et al., 2024). Another active component from pine plants, pinobanksin, which is an analog of PIN, is capable of suppressing the Notch signaling pathway, thereby halting the cell cycle and inhibiting the proliferation of lung cancer cells as well as inducing apoptosis (Win et al., 2019). PIN, a naturally occurring compound mainly obtained from pine species, is supported by a range of evidence indicating its potential anti-cancer effects. *In vitro* studies have shown that PIN, administered at concentrations ranging from 0 to 40 μM over periods of 24 and 48 h, does not markedly suppress the proliferation of normal colonic cells. Nevertheless, it has been observed to have inhibitory effects on the growth of two distinct human colon cancer cell lines, HCT116 and HT29. This is manifested by a notable and concentration-dependent rise in the percentage of cells in the S phase for both HCT116 and HT29 cell lines, coupled with a moderate enhancement in the fraction of cells in the G2/M phase specifically in HT29 cells. PIN also modulates the expression of signaling proteins associated with cell proliferation and apoptosis, particularly in HCT116 cells, it markedly elevates the expression levels of p53, Bax, cleaved caspase-3, cleaved PARP, and p21Cip1/Waf1, while concurrently reducing the expression levels of cyclin E and phosphorylated Rb (Sun et al., 2016). Hsieh et al. discovered that PIN hydrate suppresses the migration of human oral cancer cells by downregulating the p38/ERK1/2 pathway and inhibiting MMP-2 enzyme activity (Hsieh et al., 2018). PIN has been demonstrated to possess antitumor potential, yet its role in EMT has not been extensively studied until now. In this study, we discovered that PIN can effectively inhibit the EMT process in pulmonary epithelial cells. *In vitro* cell experiments showed that after 48 h of PIN treatment, the degree of EMT in lung epithelial cells decreased with the increase of PIN dose. Simultaneously, there was a significant increase in the expression of the epithelial marker E-cadherin, along with a substantial decrease in the expression of the mesenchymal marker Vimentin. This suggests that PIN exerts a clear inhibitory effect on EMT at the cellular level, with the ability to reverse the phenotypic and molecular marker alterations associated with EMT. In animal models, as evidenced by H&E and Masson’s staining of mouse lung tissue sections, the fibrosis degree in the high-dose PIN group, the pirfenidone group, and the combination group was reduced, and the destruction of alveolar structure was improved. At the same time, the expression patterns of EMT-related genes and proteins were consistent with the results of *in vitro* cellular experiments, further validating the *in vivo* inhibitory effect of PIN on pulmonary EMT. These findings imply that PIN could potentially be developed as an anti-EMT pharmaceutical, presenting a novel therapeutic avenue for pulmonary disease treatment.

The PI3K/Akt signaling pathway is widely recognized for its critical role in regulating various cellular physiological processes, including EMT. In our study, stimulation with TGF-β1 significantly activated the PI3K/Akt pathway in pulmonary epithelial cells, as evidenced by increased phosphorylation levels of both PI3K and Akt proteins. Notably, treatment with PIN effectively attenuated this activation in a dose-dependent manner, with phosphorylation levels of Akt returning to near-baseline levels. To further validate the involvement of the PI3K/Akt pathway in PIN-mediated EMT inhibition, we employed pharmacological modulators: the specific PI3K inhibitor LY294002 and the Akt activator SC79. Our results demonstrated that PIN significantly suppressed the SC79-induced upregulation of phosphorylated Akt, confirming its ability to antagonize pathway activation even in the presence of external stimulation. These findings indicate that the anti-EMT effects of PIN are, at least in part, mediated through suppression of the PI3K/Akt signaling pathway. Since this pathway is known to regulate downstream transcription factors such as Snail, which repress E-cadherin expression and promote EMT (Fang et al., 2024), we further examined the effect of PIN on Snail expression. Our results showed that PIN treatment significantly downregulated Snail and ZEB1 expression in TGF-β1-induced cells, consistent with the reversal of EMT marker expression patterns. This suggests that PIN may inhibit EMT by modulating the PI3K/Akt/Snail axis, providing a clearer mechanistic insight into its therapeutic potential.

At present, a variety of drugs and therapies targeting pulmonary EMT are in the research or clinical application phase (Wu et al., 2024; Tang et al., 2024; Singh et al., 2024). PIN offers distinctive benefits over traditional anti-fibrotic medications. Notably, in contrast to novel biological agents like anti-TGF-β antibodies, PIN is a naturally sourced compound that enjoys the advantages of widespread availability and more affordable costs. Additionally, given its multi-target action profile, the therapeutic potential of PIN, particularly when integrated with other anti-EMT pharmaceuticals or treatment modalities, is substantial. Staskiewicz et al. demonstrated that combining PIN with bortezomib induces apoptosis in human multiple myeloma cells and significantly reduces cell viability (Staskiewicz et al., 2023). Similarly, in the present study, the combination therapy group exhibited a more substantial reduction in the Pulmonary Index and greater suppression of Vimentin and α-SMA protein expression levels compared to groups treated with either drug alone. Advancing research on the synergistic use of PIN with anti-fibrotic medications featuring diverse mechanisms of action is crucial. This strategy could lead to enhanced inhibition of pulmonary EMT and fibrotic processes, optimizing therapeutic efficacy while potentially lowering the required dosage and mitigating the adverse effects associated with single-agent treatments. Additionally, the development and pharmacokinetic studies of PIN as a pharmaceutical agent remain uncharted territories, yet they hold significant promise. Delving into the intricacies of its absorption, distribution, metabolism, and excretion could unlock valuable insights, laying a robust scientific foundation for the rational clinical application of this compound. Such investigations hold the promise of developing more effective and safer clinical interventions.

## 5 Conclusion

In conclusion, our study has demonstrated that PIN effectively inhibits pulmonary EMT and is closely associated with the modulation of the PI3K/Akt signaling pathway. Both *in vitro* and *in vivo* findings reveal that PIN significantly suppresses pulmonary EMT, preserves cellular morphology, and regulates the expression of key genes and proteins involved in EMT. Additionally, PIN alleviates pulmonary fibrosis and ameliorates the pathological changes linked to EMT in animal models. Mechanistically, PIN inhibits the PI3K/Akt signaling pathway in a dose-dependent manner, with its interaction with this pathway confirmed through the use of specific activators and inhibitors. This study introduces PIN as a novel treatment strategy for pulmonary EMT, laying the foundation for further investigations into its potential applications in lung diseases.

## Data Availability

The datasets presented in this study can be found in online repositories. The names of the repository/repositories and accession number(s) can be found in the article/Supplementary Material.

## Abbreviations

IPF, Idiopathic Pulmonary Fibrosis; TGF-β1, Transforming Growth Factor-β1; EMT, Epithelial-Mesenchymal Transition; PEMT, Pulmonary Epithelial-Mesenchymal Transition; ECM, Extracellular matrix; PIN, Pinostilbene; FBS, Fetal bovine serum; PBS, Phosphate Buffer Solution; MTT, Methyl thiazolyl tetrazolium; FN, Fibronectin; α-SMA, α-Smooth Muscle Actin; ROS, Reactive Oxygen Species; BLM, Bleomycin; PDB, Protein Data Bank; BP, Biological Processes; CC, Cellular Components; MF, Molecular Functions.
