# Supplementary material for: Pinostilbene inhibits lung epithelial-mesenchymal transition and delays pulmonary fibrosis by modulating the PI3K/Akt pathway
Source: Front Pharmacol. 2025 Sep 5;16:1614546. doi: 10.3389/fphar.2025.1614546 (PMC12446271; doi:10.3389/fphar.2025.1614546)
Supplement: Supplementary file 1 [file DataSheet1.docx]

***Supplementary Material***


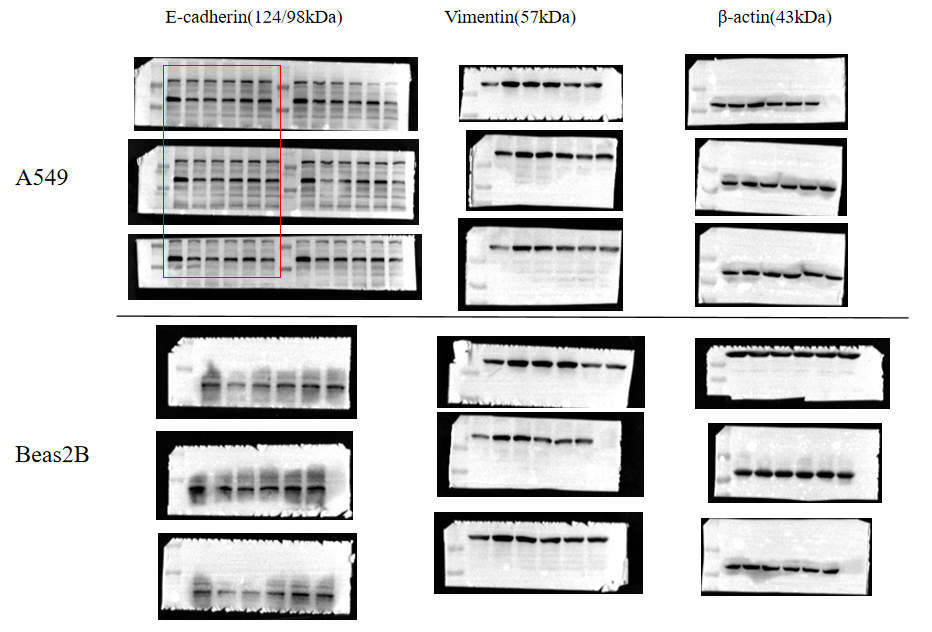


**Supplementary Figure 1.** Changes in E-cadherin and Vimentin protein expression levels in TGF-β1-induced A549 and Beas2B cells with varying concentrations of PIN intervention. n=3; compared with the control group. From left to right: Control, Model, PIN1μM, 2.5μM, 5μM, 10μM.


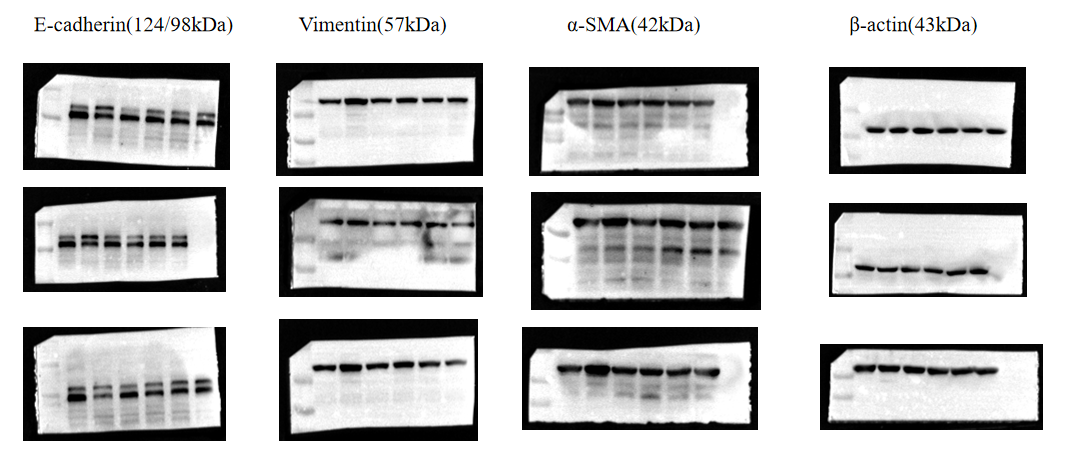


**Supplementary Figure 2.** The suppressive effect of PIN on the expression of proteins associated with bleomycin-induced pulmonary fibrosis. From left to right: Control, Model, H, L, PFD, PIN+PFD.


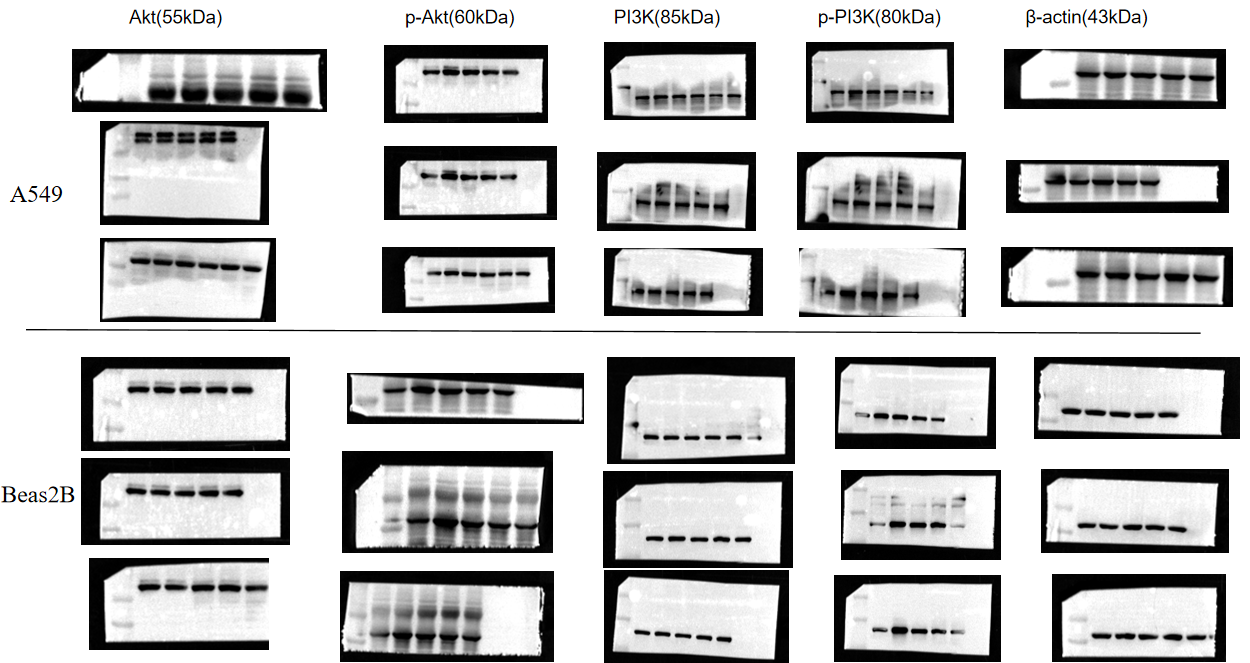


**Supplementary Figure 3.** A549 and Beas2B cells were treated with TGF-β1 (5 ng/ml) in the presence of varying concentrations of PIN (1, 2.5, and 5 μM) for a duration of 48 hours. Subsequently, cell lysates were harvested and subjected to western blot analysis to evaluate the expression levels of PI3K, p-PI3K, Akt, and p-Akt. From left to right: Control, Model, PIN1μM, 2.5μM, 5μM.


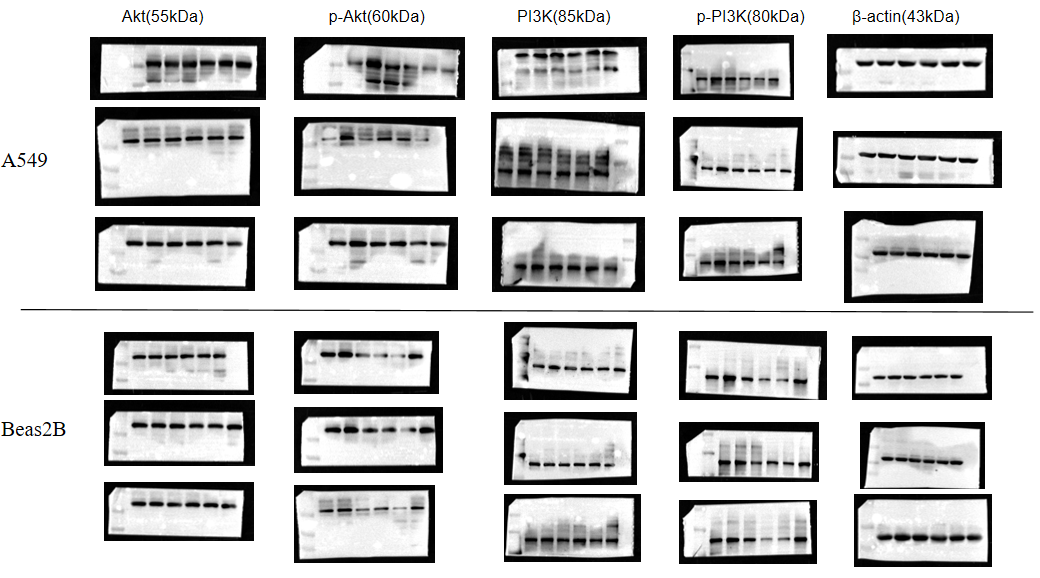


**Supplementary Figure 4.** Examination of the Impact of SC79 and LY294002 on the PI3K/AKT Pathway in A549 and Beas2B Cells. Following this treatment, cell lysates were obtained and analyzed for the expression of PI3K, p-PI3K, Akt, and p-Akt using western blotting techniques. From left to right: Control, SC79, SC79+PIN, SC+LY, SC+LY+PIN, PIN.


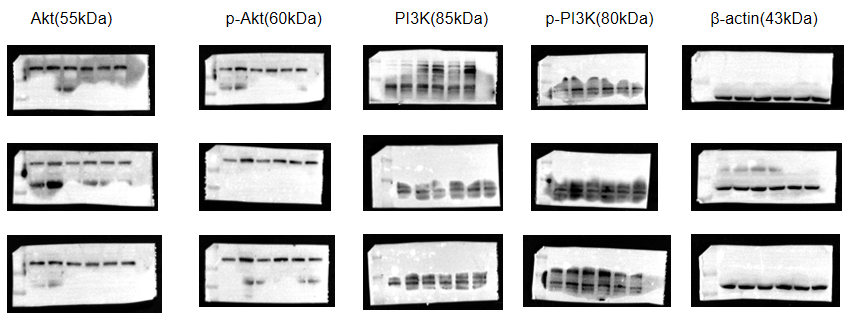


**Supplementary Figure 5.** PIN suppresses the activation of the PI3K/Akt signaling pathway induced by bleomycin in C57BL/6J male mice. From left to right: Control, Model, H, L, PFD, PIN+PFD.
